# Survival and analysis of predictors of mortality in patients undergoing replacement renal therapy: a 20-year cohort

**DOI:** 10.1186/s12882-020-02135-7

**Published:** 2020-11-23

**Authors:** Emily de Souza Ferreira, Tiago Ricardo Moreira, Rodrigo Gomes da Silva, Glauce Dias da Costa, Luciana Saraiva da Silva, Samantha Bicalho de Oliveira Cavalier, Beatriz Oliveira Silva, Heloísa Helena Dias, Luiza Delazari Borges, Juliana Costa Machado, Rosângela Minardi Mitre Cotta

**Affiliations:** 1 Department of Nutrition and Health, Federal University of Viçosa, Viçosa, Minas Gerais Brazil; 2 Department of Medicine and Nursing, Federal University of Viçosa, Viçosa, Minas Gerais Brazil; 3 Nephrology Service, Hospital São João Batista, Viçosa, Minas Gerais Brazil; 4 Department of Nutrition, Federal University of Uberlândia, Uberlândia, Minas Gerais Brazil

**Keywords:** Hemodialysis, Renal replacement therapy, End-stage renal disease, Chronic kidney disease; biochemical parameter

## Abstract

**Background:**

optimal management of end-stage renal disease (ESRD) in hemodialysis (HD) patients should be more studied because it is a serious risk factor for mortality, being considered an unquestionable global priority.

**Methods:**

we performed a retrospective cohort study from the Nephrology Service in Brazil evaluating the survival of patients with ESRD in HD during 20 years. Kaplan-Meier method with the Log-Rank and Cox’s proportional hazards model explored the association between survival time and demographic factors, quality of treatment and laboratory values.

**Results:**

Data from 422 patients were included. The mean survival time was 6.79 ± 0.37. The overall survival rates at first year was 82,3%. The survival time correlated significantly with clinical prognostic factors. Prognostic analyses with the Cox proportional hazards regression model and Kaplan-Meier survival curves further identified that leukocyte count (HR = 2.665, 95% CI: 1.39–5.12), serum iron (HR = 8.396, 95% CI: 2.02–34.96), serum calcium (HR = 4.102, 95% CI: 1.35–12.46) and serum protein (HR = 4.630, 95% CI: 2.07–10.34) as an independent risk factor for the prognosis of survival time, while patients with chronic obstructive pyelonephritis (HR = 0.085, 95% CI: 0.01–0.74), high ferritin values (HR = 0.392, 95% CI: 0.19–0.80), serum phosphorus (HR = 0.290, 95% CI: 0.19–0.61) and serum albumin (HR = 0.230, 95% CI: 0.10–0.54) were less risk to die.

**Conclusion:**

survival remains low in the early years of ESRD treatment. The present study identified that elevated values of ferritin, serum calcium, phosphorus, albumin, leukocyte, serum protein and serum iron values as a useful prognostic factor for the survival time.

## Background

Chronic Kidney Disease (CKD) and its evolution to end-stage renal disease (ESRD) is increasingly recognized as a serious risk factor for mortality, being considered an unquestionable global public priority [1–4]. In 2016 CKD was the 13th in the list of causes of death, with projection that in 2040 it will be the 5th leading cause of death worldwide [2, 5].

Worldwide, the total number of individuals with acute kidney injury, CKD and Renal Replacement Therapy (RRT) exceeds 850 million, a figure that is double the estimated number of people with diabetes worldwide [6]. Data from the 2017 Annual Report of the European Renal Association - European Dialysis and Transplant Association (ERA-EDTA) [7] report that 83.311 individuals from all countries of Europe started RRT in 2016. At the end of the same year, the total number of individuals requiring RRT was 564.638, with more than 80% being on hemodialysis (HD). In Brazil, also in 2017, the total number of patients on chronic dialysis was equivalent to 162.583, with 91.8% of patients undergoing HD and 85% of this treatment modality funded by the Unified Health System (SUS) [8, 9].

HD is the major treatment modality for RRT worldwide, and it is also the one with the highest mortality rates, followed by kidney transplantation and peritoneal dialysis [4, 10, 11]. For these reasons, one of the most worrying outcomes of CKD is ESRD, in which there is a need for RRT.

Identifying the prognostic factors that are associated to and worsen ESRD certainly helps to reduce morbidity and mortality, especially in the first year of patients undergoing HD [4, 12]. For this, it is necessary to analyze the dynamics of biochemical parameter and different factors in the survival time of patients at baseline and for a long follow-up period, which few studies focus on doing [13]. Most epidemiological studies evaluate the prognostic value of a single complication to relate it to survival. However, considering them in isolation is inconsistent in practice, since patients have several irregularities concomitantly.

Thus, considering that the relationship of some biochemical parameter and baseline conditions in the first year of HD leads to lower short and long-term survival, the present study sought to investigate the survival time and the prognosis factors associated with this time in patients with ESRD undergoing HD.

## Methods

### Study design and population

This is a retrospective cohort study, which evaluated the data of 422 patients on HD, over a period of 20 years (January 1998 to December 2018).

All 463 patients registered at the Nephrology Service were investigated. All patients were followed retrospectively until death or censorship (end of follow-up, loss of follow-up, transfer or kidney transplant). Of the 463 patients, 22 were censored because received a transplant and 19 were censored because they were transferred or for loss of follow-up. Thereby, 422 patients had all the data available for analysis. The monitoring ended on December 31, 2018.

Data were obtained from medical records of patients undergoing treatment and from the computerized record system of the Nephrology Service in the municipality of Viçosa, Brazil, where the study was conducted and which works with a total of three sessions per week in HD with an average of 4 h each. This Nephrology Service, located in a hospital, is the only one located in the region and covers the entire Health Microregion of Viçosa, which has nine neighboring municipalities, totalizing 138.336 inhabitants.

For this study, we included consecutive patients with ESRD at any time during the study follow-up, with a minimum treatment time of one month, age ≥ 18 years and with all available baseline data. To be diagnosed with CKD, patients would have to show an irreversible decline in kidney function, that is, a Glomerular Filtration Rate (GFR) ≤ 20 ml/min/1.73m^2^ estimated by the CKD-EPI formula for more than a month. The diagnosis of underlying kidney disease was based on clinical, laboratory and radiological characteristics.

### Demographic and clinical data of the study patients

The main baseline data set collected (one month after starting hemodialysis) were: sex, age, race, occupation, marital status, length of treatment, hemodialysis characteristics and the main cause of ESRD.

After the initiation of therapy, information on biochemical investigation was collected, including: pre and post-dialysis urea (mg/dl), serum creatinine (mg/dl), serum calcium (mg/dl), serum phosphorus (meq/lit), glucose (mg/dl), serum potassium (meq/dl), hemoglobin (g/dl), hematocrit (%), erythrocyte (g/dl), leukocyte (μg/l), serum albumin (g/dl), transferrin saturation (%), ferritin (μg/l), iron serum (μg/l), alkaline phosphatase (U/l), serum protein (g/dl), globulin (g/dl), parathyroid hormone (PTH) (pg/ml), aluminum (μg/l), cholesterol (mg/dl), triglycerides (mg/dl), calcium x phosphorus ratio (mg/dl), PRU (rheumatological profile) and nPCR (normalized protein catabolism rate) (g/dl). The analyzes were made at the baseline, that is, when the patients started treatment.

The cutoff points for each biochemical parameter were defined in terciles or according to the recommendations for patients undergoing HD [14–16].

### Details of hemodialysis

All patients were dialyzed using standard bicarbonate hemodialysis, performed three times a week with an average duration of 4 h. Dialysis machines of individual proportion were used with water treated by reverse osmosis. Volumetric ultrafiltration control was available on all machines. The standard dialysate flow rate was 500 ml/min and blood flow rates were directed according to the patient’s needs. Dialyzer reuse was performed uniformly using automated methods.

The adequacy of dialysis was determined using the unique Kt/V pool (spKt/V) obtained by the Fresenius 4008S HD machine (Fresenius Medical Care AG, Bad Homburg, Germany) equipped with an *online clearance* monitor, in the same day of blood collection. Based on the guidelines of the Kidney Disease Outcome Quality Initiative (KDOQI) 2015 [16] in HD, a Kt/V ≥ 1.2 is considered as the minimum dialysis dose for three times a week. Therefore, the Kt/V cutoff point of 1.2 was used to study the effect of varying doses of dialysis on survival for this study.

### Statistical analysis

The percentage, mean ± standard deviation (SD), or median and interquartile range (IQR) were used to describe and summarize the baseline data. We tabulated characteristics of patients at the time of their first record of the HD session. The primary objective of the analysis was death.

The initial event (E0) was characterized by the date on which the patient was admitted for HD treatment; the second event (E1), the date on which he stopped performing the procedure for any of the outcomes described above. The outcome of all patients in terms of survival and mortality, as well as the prognostic factors that had the greatest impact on patient survival were analyzed. The strength of association was measured by the Hazard Ratio (HR).

The Kaplan-Meier method was used to calculate the cumulative survival rate and the Log-Rank test, to extract factors that impacted the survival rate and examine the data. Log-Rank test is most effective at the beginning and in the middle of following the survival curve. In addition, Cox’s proportional hazards model was used with HD treatment as a time-dependent exposure to calculate risk rate and the influence of several clinical and demographic variables on patient survival.

The selection of variables was based on scientific evidence, clinical importance and the result of univariate analysis, so that variables with a *p*-value ≤0.200 were determined to be significant and included in the multivariate regression model. To find the optimal survival regression model, we used backward selection procedure by Wald’s test, which starts from a complete model and removing the weakest predictors from the candidate list one by one until only statistically significant (*p* ≤ 0.05) predictors remain (the selection cut-off value was from default as well as the importance of clinical concern) [17, 18]. The confidence interval (CI) adopted was 95% and a *p* < 0.05 was used for statistical significance.

The proportional hazards assumptions were tested using time-dependent covariate analysis in the adjusted models in SPSS and Kaplan Meier curves and have not been violated.

To avoid the problem of overfitting owing to the number of outcomes, we performed bootstrapping validation, in order to determine the CIs for estimating β in the Cox proportional hazard regression (1000 bootstraps) [19, 20]. The significance of the differences among different methods was determined with the use of the signed-rank test for bias and the bootstrap method for the interquartile range from the 1000 bootstrap samples. All analyzes were performed using IBM SPSS Statistics 23.0.

## Results

Beta coefficients of the independent variables obtained by multivariate Cox proportional hazards analysis were assessed using 1000 bootstrap resamples. No significant difference between original beta and bootstrapped coefficients was observed.

Table 1 shows some characteristics of the patient’s baseline and the laboratory values ​​measured. The total number of patients was 422. The average age at the beginning of HD was 64.02 ± 15.21 years (mean ± standard deviation), with 132 (31.3%) patients started HD before age 60 and 223 (52.7%) are men. Hypertensive renal disease with renal failure was responsible for 33.4% of the cases with varying etiology. The recommended Kt/v values ​​ (≥ 1.2) were found in 237 (56.2%) patients and the average GFR, calculated using the CKD-EPI formula, was 10.13 mL/min/1.73 m^2^.

**Table 1:** Baseline characteristics and laboratory values of patients on hemodialysis

Baseline characteristics of the patients	Number of patient (%)
**Age in years (mean ± SD)**	64,02 (15,21)
**Age (year)**	
< 60	132 (31,3)
60–69	90 (21,3)
70–79	101 (23,9)
≥ 80	55 (13,0)
**Demographic features**	
**Sex**	
Male	223 (52,8)
Female	199 (47,2)
**Ethnicity**	
White	207 (49,1)
Black	78 (18,5)
Brown	88 (20,9)
**Occupation**	
Retired	127 (30,1)
From home	85 (20,1)
Formal work	58 (13,7)
Informal work	29 (6,9)
Rural worker	54 (12,8)
Student	7 (1,7)
**Civil status**	
Married	211 (50,0)
Single	57 (13,5)
Widow	85 (20,1)
Separated	20 (4,7)
**Primary causes of end-stage renal disease**	
Glomerular Disorder in Diabetes Mellitus	98 (23,2)
Chronic obstructive pyelonephritis	8 (1,9)
Hypertensive kidney disease with CKD	141 (33,4)
Others	42 (10,0)
**Dialysis session indices**	
Kt/v (urea) (mean ± SD)	1,67 (4,24)
0 a 1,19	139 (32,9)
≥ 1,20	237 (56,2)
Time of hemodialysis (months)(mean ± SD)	52,06 (49,91)
GFR (mean ± SD)	10,13 (6,82)
**Laboratory Values (mean ± SD)**	
Post urea (mg/dl)	41,90 (25,20)
Creatinine (mg/dl)	6,47 (3,10)
Calcium (mg/dl)	8,75 (1,57)
Phosphorus (meq/lit)	5,01 (1,86)
Glucose (mg/dl)	124,46 (59,06)
Potassium (meq/lit)	4,83 (0,96)
Red blood cell (g/dl)	100, 22 (151,03)
Hemoglobin (g/dl)	8,97 (1,73)
Hematocrit (%)	27,79 (5,08)
Leukocyte (μg/l)	9,30 (41,47)
Albumin (g/dl)	3,88 (3,18)
Transferrin Saturation (%)	30, 61 (23,66)
Ferritin (μg/l)	480,26 (644,41)
Serum iron (μg/l)	70,00 (43,29)
Alkaline phosphatase (U/l)	205,61 (156,19)
Proteins (mg/dl)	7,38 (11,38)
Globulin (g/dl)	4,10 (19,99)
PTH (pg/ml)	233,76 (290,51)
Aluminum (μg/l)	11,75 (15,70)
Cholesterol (mg/dl)	165,72 (42,25)
Triglyceride (mg/dl)	163,86 (107,84)
PRU	65,83 (12,06)
nPCR (mg/dl)	1,55 (5,13)
Calcium x Phosphorus (mg/dl)	46,21 (49,15)
Pre urea (mg/dl)	116,69 (42,66)

*Abbreviations*: *CKD* chronic kidney disease, *SD* standard deviation, *PTH* parathyroid hormone, *nPCR* normalized catabolic protein rate, *PRU* rheumatological profile

Regarding biochemical parameter, the averages of serum calcium, hemoglobin, hematocrit, serum albumin and pre-dialysis urea were below recommendations for these patients, while PTH and triglycerides were above recommended and the other exams, within the normal range [14–16].

**Fig. 1:**
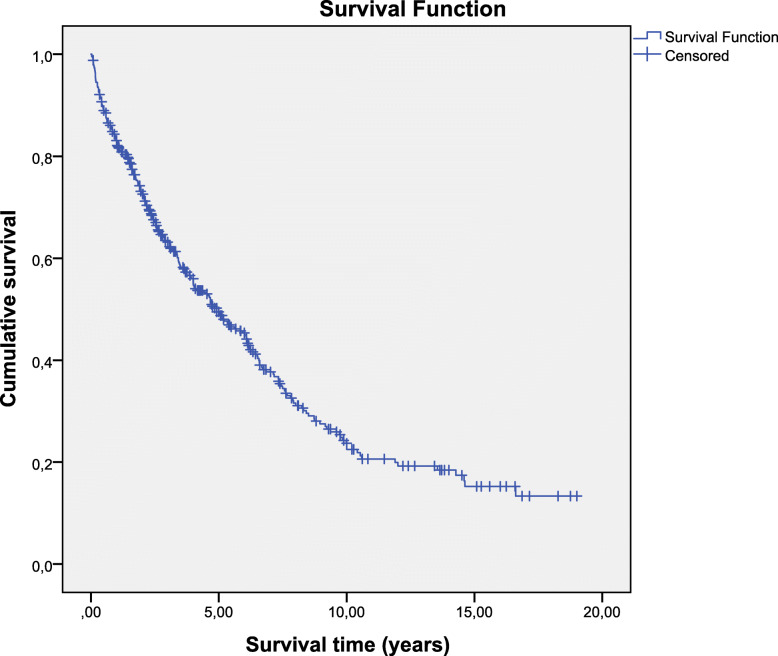
- Kaplan–Meier survival curves of chronic hemodialysis

Figure 1 shows cumulative survival rate for all patients. The number of patients who died was 254 (60.2%); therefore, 39.8% of the survival time was censored. The mean survival time is 6.79 ± 0.37 years (mean ± standard deviation) and 95% CI 6.06–7.51. The survival rate was 82.3% in the first year, 49.1% in 5 years, 22.5% in 10 years and 13.3% at the end of the follow-up. The median survival time was 4.92 (3.914–5.924) (median ± interquartile range); this means that 50% of individuals have survived for at least 4.92 years.

Cumulative survival rate calculated by Kaplan Meier method is displayed according to each factor, and the Log-rank tests were used to compare these factors through univariate analysis (Tables 2 and 3).

**Table 2:** Influence of sociodemographic factors and baseline dialysis on the survival time

Factors	Mean survival time (year) (DP)	95% CI (years)	*p*-value^a^
**Age (year)**			
< 60	7,506 (0,683)	6,167 – 8,845	0,048
≥ 60	5,804 (0,399)	5,022 – 6,586	
**Sex**			
Male	6,529 (0,448)	5,651 – 7,406	0,867
Female	6,780 (0,547)	5,708 – 7,853	
**Ethnicity**			
White	7,021 (0,529)	5,984 – 8,058	
Black	5,411 (0,725)	3,990 – 6,833	0,300
Brown	6,419 (0,742)	4,964 – 7,874	
**Occupation**			
Retired	5,813 (0,528)	4,778 – 6,848	
From home	7,058 (0,876)	5,340 – 8,776	
Formal work	8,234 (1128)	6,023 – 10,444	0,800
Informal work	3,616 (0,645)	2,351 – 4,881	
Rural worker	7,136 (0,948)	5,277 – 8,994	
Student	5,861 (1570)	2,784 – 8,938	
**Civil status**			
Married	7,241 (0,523)	6,216 – 8,266	
Single	5,490 (0,781)	3,958 – 7,021	0,115
Widow	5, 303 (0,647)	4,034 – 6,572	
Separated	7,558 (1785)	4,061 – 11,056	
**Primary causes of end-stage renal disease**			
Glomerular Disorder in DM	7,485 (0,726)	6,062 – 8,909	
Chronic obstructive pyelonephritis	11,883 (2896)	6,207 – 17,559	0,135
Hypertensive kidney disease with CKD	8,227 (0,673)	6,907 – 9,547	
Others	5,688 (0,946)	3,834 – 7,542	
**Kt/v**			
**≤** 1,19	7,015 (0,638)	5,764 – 8,266	0,563
≥ 1,20	6,723 (0,547)	5,651 – 7,794	

Notes^: a^Log-rank test

*Abbreviations*: *CKD* chronic kidney disease, *DM* diabetes mellitus, *SD* standard deviation

**Table 3:** Influence of biochemical parameter on the survival time of patients on hemodialysis

Factors	Mean survival time (year) (SD)	95% CI (years)	*p*-value^a^
**Laboratory Values**			
Post urea (mg/dl)			
≤ 29,00	5,411 (0,548)	4,337 – 6,485	0,006
29,01 – 47,00	7,594 (0,640)	6,339 – 8,849	
≥ 47,01	7,335 (0,655)	6,051 – 8,618	
**Creatinine (mg/dl)**			
≤ 4,80	5,687 (0,649)	4,414 – 6,960	0,030
4,81 – 7,20	7,008 (0,753)	5,532 – 8,485	
≥ 7,21	7,672 (0,719)	6,262 – 9,082	
**Calcium (mg/dl)**			
≤ 8,99	6,803 (1226)	4,399 – 9,206	0,154
9,00 – 11,00	6,597 (0,598)	5,425 – 7,768	
≥ 11,01	4,390 (0,896)	2,635 – 6,146	
**Phosphorus (meq/lit)**			
≤ 4,49	5,676 (0,533)	4,632 – 6,721	0,047
4,50 – 6,00	7,626 (0,759)	6,138 – 9,114	
≥ 6,01	7,118 (0,891)	5,371 – 8,865	
**Glucose (mg/dl)**			
≤ 99,99	7,155 (0,603)	5,973 – 8,337	0,768
100,00 – 125,99	6,423 (0,776)	4,903 – 7,944	
≥ 126,00	6,794 (0,638)	5,545 – 8,044	
**Potassium (meq/lit)**			
≤ 4,40	6,263 (0,675)	4,940 – 7,585	0,530
4,41 – 5,20	6,981 (0,686)	5,636 – 8,326	
≥ 5,21	6,747 (0,652)	5,469 – 8,025	
**Red blood cell (g/dl)**			
≤ 3,00	6,173 (0,393)	5,404 – 6,943	0,434
3,01 – 4,00	6,656 (0,705)	5,273 – 8,038	
≥ 4,01	6,328 (0,531)	5,287 – 7,369	
**Hemoglobin (g/dl)**			
≤ 8,30	6,716 (0,658)	5,427 – 8,004	0,991
8,31 – 9,70	6,458 (0,681)	5,123 – 7,793	
≥ 9,71	6, 288 (0,658)	5,299 – 7,877	
**Hematocrit (%)**			
≤ 25,30	6,792 (0,690)	5,439 – 8,145	0,896
25,31–29,80	6,485 (0,698)	5,117 – 7,852	
≥ 29,81	6,672 (0,645)	5,408 – 7,937	
**Leukocyte (μg/l)**			
≤ 5,80	7,455 (0,716)	6,052 – 8,858	0,059
5,81 – 7,70	5,571 (0,497)	4,597 – 6,546	
≥ 7,71	7,592 (0,708)	6,204 – ,980	
**Albumin (g/dl)**			
≤ 3,50	6,256 (0,640)	5,002 – 7,510	0,059
3,51 – 4,00	7,921 (0,670)	6,608 – 9,234	
≥ 4,01	6,500 (0,587)	5,350 – 7,650	
**Ferritin (**μg**/l)**			
≤ 178,40	5,872 (0,569)	4,757 – 6,986	0,049
178,41–494,00	8,265 (0,740)	6,815 – 9,715	
≥ 494,01	6,050 (0,522)	5,026 – 7,073	
**Serum iron (μg/l)**			
≤ 49,99	6,591 (0,630)	5,356 – 7,826	0,065
50,00–150,00	7,140 (0,486)	6,187 – 8,093	
≥ 150,01	3,757 (0,887)	2,019 – 5,495	
**Alkaline phosphatase (U/l)**			
≤ 131,00	6,740 (0,860)	5,055 – 8,425	0,636
131,01–221,00	7,437 (0,653)	6,157 – 8,716	
≥ 221,01	6,275 (0,472)	5,351 – 7,199	
**Proteins (mg/dl)**			
≤ 6,30	6,865 (0,640)	5,609 – 8,120	0,122
6,31–7,00	7,403 (0,673)	6,084 – 8,722	
≥ 7,01	6,174 (0,550)	5,096 – 7,252	
**Globulin (g/dl)**			
≤ 2,72	6,779 (0,571)	5,660 – 7,898	0,775
2,73–3,40	6,280 (0,516)	5,268 – 7,292	
≥ 3,41	7,345 (0,770)	5,836 – 8,855	
**PTH (pg/ml)**			
≤ 2,72	6,911 (0,649)	5,638 – 8,184	0,682
2,73–3,40	7,107 (0,684)	5,766 – 8,448	
≥ 3,41	6,572 (0,473)	5,644 – 7,500	
**Aluminum (**μg**/l)**			
≤ 5,90	7,533 (0,774)	6,016 – 9,051	0,307
5,91–12,40	8,625 (0,790)	7,078 – 10,173	
≥ 12,41	7,005 (0,567)	5,893 – 8,116	
**Cholesterol (mg/dl)**			
≤ 145,00	7,798 (0,662)	6,501 – 9,095	0,320
146,00–179,00	9,229 (0,825)	7,612 – 10,847	
≥ 180,00	8,179 (0,791)	6,629 – 9,730	
**Triglyceride (mg/dl)**			
≤ 110,00	7,836 (0,655)	6,553 – 9,120	0,903
110,01–170,00	8,797 (0,868)	7,095 – 10,498	
≥ 170,01	7,859 (0,602)	6,679 – 9,040	
**PRU**			
≤ 62,32	7,514 (0,685)	6,172 – 8,856	0,076
62,33–70,97	7,630 (0,634)	6,389 – 8,872	
≥ 70,98	5,441 (0,517)	4,427 – 6,454	
**nPCR (mg/dl)**			
≤ 0,91	6,959 (0,785)	5,419 – 8,498	0,043
0,92–1,33	8,061 (0,634)	6,819 – 9,303	
≥ 1,34	6,523 (0,596)	5,354 – 7,691	
**Calcium x Phosphorus (mg/dl)**			
≤ 34,24	5,782 (0,578)	4,649 – 6,915	0,141
34,25–49,24	7,257 (0,745)	5,797 – 8,718	
≥ 49,25	7,006 (0,707)	5,619 – 8,392	
**Pre urea (mg/dl)**			
≤ 95,00	5,796 (0,643)	4,536 – 7,055	0,121
95,01–132,00	7,273 (0,777)	5,750 – 8,796	
≥ 132,01	7,239 (0,690)	5,886 – 8,592	

Notes^: a^Log-rank test

*Abbreviations*: *CKD* chronic kidney disease, *DM* diabetes mellitus, *SD* standard deviation

The univariate analysis showed, between the socio-demographic variables, there is a significant difference (*p* < 0.20) in age (< 60 years and ≥ 60 years), marital status and primary cause of ESRD. On the other hand, the analysis didn’t show statistical difference between men and women.

In the biochemical parameter, the variables with significant differences were: pre-dialysis urea, post-dialysis urea, serum creatinine, serum phosphorus, serum albumin, ferritin, serum iron, serum protein, PRU, nPCR, calcium x phosphorus ratio, serum calcium and leukocytes (Table 3).

As can be seen in Table 4, in this multivariate analysis it is considered that, among the clinical and demographic variables, those that significantly influenced patient survival were: the primary cause of ESRD, leukocytes, ferritin, serum iron, serum albumin, serum protein, serum calcium and serum phosphorus.

**Table 4:** Factors affecting survival time

Factors	HR (95% CI)	*P* – value*
Primary causes of end-stage renal disease		
Glomerular Disorder in DM	1,00	
Chronic obstructive pyelonephritis	0,085 (0,010 – 0,744)	0,026
Hypertensive kidney disease with CKD	1,153 (0,589 – 2,260)	0,678
Others	3,589 (1,577 – 8,166)	0,002
Leucocytes (μg/l)		
≤ 5,80	1,00	
5,81–7,70	2,665 (1,387 – 5,119)	0,003
≥ 7,71	1,435 (0,687 – 2,997)	0,336
Ferritin (μg/l)		
≤ 178,40	1,00	
178,41–494,00	0,350 (0,177 – 0,692)	0,003
≥ 494,01	0,392 (0,192 – 0,799)	0,010
Protein (mg/dl)		
≤ 6,30	1,00	
6,31–7,00	1,574 (0,694 – 3,574)	0,278
≥ 7,01	4,630 (2,073 – 10,341)	0,001
Calcium (mg/dl)		
≤ 8,99	1,00	
9,00–11,00	1,202 (0,499 – 2,896)	0,682
≥ 11,01	4,102 (1,351 – 12,457)	0,013
Phosphorus (meq/lit)		
≤ 4,49	1,00	
4,50–6,00	0,873 (0,460 – 1,657)	0,678
≥ 6,01	0,290 (0,138 – 0,609)	0,001
Serum iron (μg/l)		
≤ 49,99	1,00	
50,00–150,00	0,602 (0,330 – 1,099)	0,098
≥ 150,01	8,396 (2,016 – 34,959)	0,003
Albumin (g/dl)		
≤ 3,50	1,00	
3,51–4,00	0,422 (0,182 – 0,979)	0,044
≥ 4,01	0,230 (0,097 – 0,541)	0,001

Notes: * Multivariate analysis - Cox regression

*Abbreviations*: *CKD* chronic kidney disease, *DM* diabetes mellitus

**Fig. 2:**
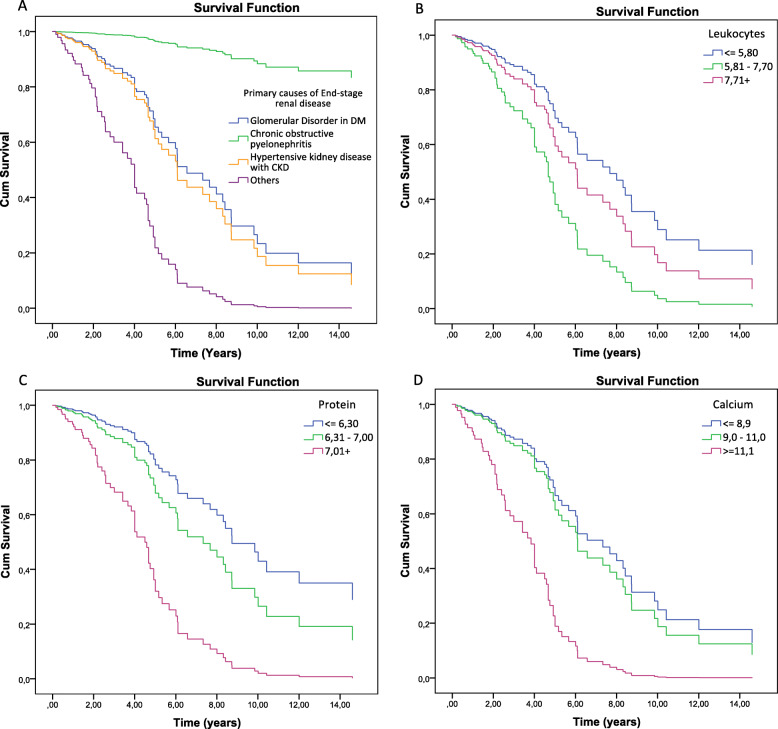
Cox regression and the risk factors; ESRD, leukocyte, serum protein, serum calcium

Patients who presented chronic obstructive pyelonephritis as a cause of ESRD resulted in better prognosis of survival time when compared to glomerular disorder in diabetes mellitus (HR = 0.085, 95% CI: 0.010–0.744). Patients with other causes of the disease, such as urinary tract disorders, hypertensive nephrosclerosis and non-specific chronic renal failure, resulted in worst prognosis of survival when compared to glomerular disorder in diabetes mellitus (HR = 3.599, 95% CI 1.577–8.166) (Table 4 and Fig. 2a).

**Fig. 3:**
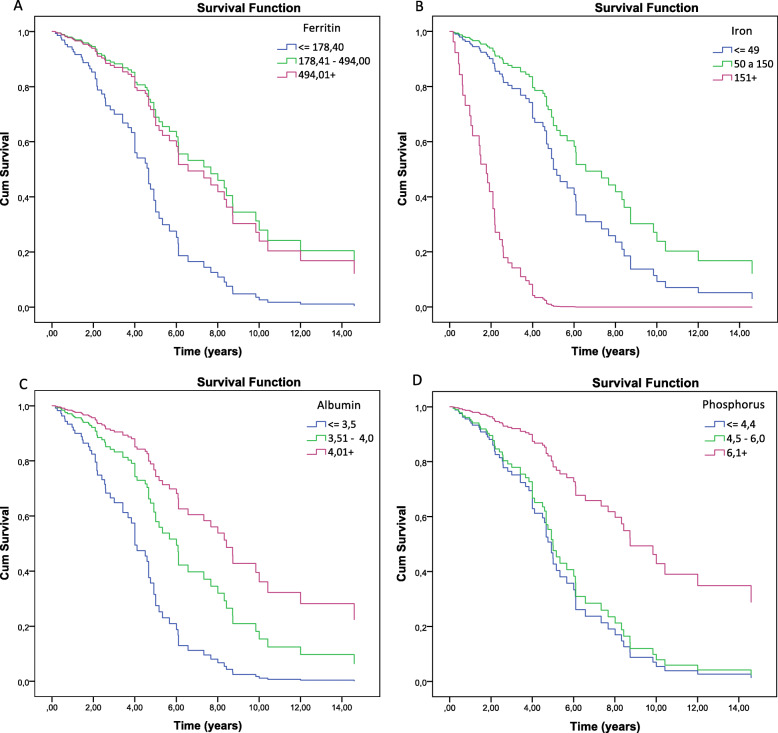
Cox regression and risk factors; ferritin, serum iron, serum albumin, serum phosphorus

Prognostic factors of survival were explored suggesting that patients with adequate values ​​of ferritin (178.4 μg/l ≥ 494.0 μg/l) and serum albumin (≥ 4.01 g/dl), resulted in better prognosis, with respectively, 2.85 (HR = 0.392, 95% CI 0.192–0.799) and 4.34 (HR = 0.230, 95% CI 0.097–0.541) compared to values ​​below the recommendation for HD patients (Table 4 and Fig. 3a and c).

In contrast, patients with elevated serum iron values ​​(≥ 150.0 μg / l), serum protein (≥ 7.01 mg/dl) and serum calcium (≥ 11.01 mg/dl) and normal values ​​of leukocytes (5.81 μg/l to 7.70 μg/l), have, respectively, a low survival prognosis: 8.396 (95% CI 2.016–10.341), 4.630 (95% CI 2.073–10.341), 4.102 (95% CI 1.351–12.457) and 2665 (95% CI 1.387–5.119) compared to patients with values ​​below the recommended (Table 4; Fig. 2b, c and d and Fig. 3b). Patients with serum phosphorus above recommended (≥ 6.01 meg / lit) have an HR of 0.290 (95% CI 0.138–0.609) (Table 4; Fig. 3d).

## Discussion

In both scientific and clinical practice, prediction aims at accurately predicting the risk of an outcome using multiple predictors collectively, where the final prediction model is usually based on statistically significant, but not necessarily causal, associations in the data at hand [21]. Choice in a dataset have impact of the predictimand of patients with ESRD, so, defining the estimand is equally important in prediction research as in causal inference [22].

Our study provided a set of indicators and parameters that showed to be was an important prognostic factor for survival of patients in the first year of treatment in HD, such as: basic cause of ESRD, serum phosphorus, serum calcium, leukocytes, serum protein, serum albumin, serum iron and ferritin.

The median survival time was 6.79 ± 0.37 years (mean ± standard deviation), and 254 (60.2%) patients died. In the study by Nyuyen et al. (2017) [23] the survival time was 5.27 ± 0.31 years (mean ± standard deviation), in the study by Browne et al. (2014) [1] the death rate was 60%, in the study by Ebhahimi et al. (2019) [4] of 52% and in the study by Chandrashekar et al. (2014) [24] 19.8% over 2 years. These periods are relatively short when compared to US rapporteurs [24, 25]. Although our study has similar values ​​to cohorts performed in other regions, there is variability in the survival rate of HD patients in different countries, therefore, these numbers cannot be compared to simple terms.

Regarding survival rate, it was 82.3% in the first year, 49.1% in 5 years, 22.5% in 10 years and 13.3% at the end of the follow-up. Nyuyen (2017) [23] found in her cohort an 85% survival rate at 1 year, 58% at 5 years and 20% at 10 years. Belino et al. (2017) [26] found a rate of 93.4% at the end of the second year of treatment and Teixeira et al., (2015) [27], found a survival rate in the first similar to our study: 84.71%. In the study by Chandrashekar (2014) [24], of the 19.8% death rate, a significant number of these deaths occurred in the first six months of treatment.

Comparing survival with that of normal population, adjusting mortality from all causes, is 6.1 to 7.8 times higher in HD patients than normal individuals, especially in the first year of treatment [28]. The high mortality rate in the first year of treatment can be justified by the lack of vascular access for hemodialysis, the age of patients when starting treatment and the lack of early diagnosis of CKD, when the first risk factors start to appear [26, 28, 29]. In addition, unplanned start of hemodialysis requires the use of catheters as a vascular access, which is proven to be one of the causes of the lowest survival rate in patients due to the urgency that this procedure requires, making it impossible for the patient to adapt adequately [30].

Currently, it is estimated that 425 million people worldwide are diagnosed with diabetes mellitus and it is estimated that more than 20 and up to 40% of diabetic patients develop CKD, with a significant number of people who develop CKD [30–33]. It is currently the leading cause of ESRD in developed countries [34]. In our study, individuals with glomerular disorder in diabetes mellitus had lower survival (*p* ≤ 0.001), which calls our attention to the monitoring of this disease as a prognostic factor for CKD development and subsequently ESRD.

In the analysis of the patient’s blood count, only the leukocyte was an independent prognostic factor for patient survival and its normal to high values (5.81–7.70 and ≥ 7.71) had an HR of, respectively 2.665 (95% CI 1.387–5.119) and 1.435 (95% CI 0.687–2.997). The study by Ebrahimi et al. (2019) [4], confirmed our findings by indicating that as the level of leukocyte count increases, the survival time of patients undergoing HD decreases. A study carried out in Taiwan, showed that the total leukocyte count predicts all causes and cardiovascular mortality in just one year [35], while in another study carried out in the United States, the reduction in the lymphocyte count are risk factors for mortality [36].

These findings can be justified by the fact that patients with ESRD on HD exhibit a chronic inflammatory state with a consequent increase in oxidative stress and impaired immune response [37–39]. The dialysis procedure itself contributes strongly to these adverse conditions due to exposure to synthetic materials contained in the dialysis filters and tubes, which favors a repetitive contact between circulating leukocytes and dialysis membranes and, clinically, this repetitive activation of cells can contribute to the increase in morbidity and mortality associated with HD [40, 41].

This predominant inflammatory condition of HD patients also explains the results we found in our study in relation to serum protein (95% CI 2.073–10.341). Elevated protein catabolism is common in patients with ESRD and its underlying etiology includes other complications, such as metabolic acidosis, uremia, systemic inflammation, anemia and other factors that further worsen kidney function and provide greater risks of mortality [42, 43].

Still on proteins, serum albumin is an independent and powerful prognostic index for HD patients, with evidence that at low levels, it also predicts low survival in ESRD [4]. We found a significant difference in the values of serum albumin, in which individuals in HD with serum albumin ≥4.0, had an HR of 0.230 (95% CI 0.097–0.541). Many studies have shown that hypoalbuminemia is a good predictor of mortality.

The 10-year cohort of Kato, Castro and Natarajan (2013) [45] indicated a high risk of mortality also in HD patients with serum albumin levels ≤3.8 g/dl; Teixeira et al. (2015) [27] also found that albumin is related to low survival; Msaad et al. (2019) [44] reported that 77.27% of deceased patients had low albumin and Ebhahimi et al. (2019) [4] demonstrated that for each unit (in g/dl) of increase in serum albumin, the survival time for HD patients increased by approximately 23%.

In addition, hypoalbuminemia is considered a marker of malnutrition and a strong predictor of death in these patients. However, serum albumin levels should be used with caution as nutritional markers for these patients, because low levels of this protein in HD patients are also associated with malnutrition, inflammation and, consequently, with other complications such as anemia secondary to ESRD [42, 45, 46].

Patients with ESRD have a series of hematopoietic abnormalities, most commonly, anemia, which is a multifactorial disease that affects almost all patients undergoing HD and is related to the risk of early death [27, 46–48]. HD patients are predisposed to iron deficiency due to residual blood loss during treatment [49, 50].

Serum ferritin is also an established marker for detecting absolute serum iron deficiency in patients on dialysis [48]. The 2012 KDIGO [16] guidelines for anemia management recommend that you assess iron stores by measuring ferritin at least every three months, which was done in our study. We found that patients with adequate ferritin values and with high serum iron might be associated with a poor prognosis of survival time in the treatment of HD.

Regarding calcium, individuals with high values (≥ 11.01 mg/dl) had an HR of 4.102 (95% CI 1.351–12.457). In contrast, patients with high serum phosphorus (≥ 6,01 meq/lit) had an HR of 0,290 (95% CI 0.138–0.609). Phosphorus excretion is reduced with the kidney damage that HD users have, which leads to increased levels of phosphorus in the blood. When there is an imbalance of phosphorus, what happened in our study, it leads to a significant loss of calcium and, consequently, to a debilitating bone disease [51]. Although in the advanced stages most patients present hyperphosphatemia, studies show [52] that the reduction in serum phosphorus improves patient survival, which was not found in our study. This can be explained by the fact that as GFR decreases, serum phosphorus levels increase [53].

However, a positive calcium balance arises easily because the intestinal absorption of calcium is greater than the renal excretion capacity, therefore, the excretion of this mineral in the kidneys decreases and does not increase [52]. In addition, because hypocalcemia is common in ESRD, routine calcium supplementation exists. However, high levels are associated with undesirable and harmful effects, such as vascular calcification, cardiovascular disease and mortality [53]. As with our findings, Inaguma et al. (2017) [53] evidenced in their multivariate analysis that high serum calcium levels are an independent risk factor for a poor survival prognosis.

Our study has several strengths, one of which is the complete coverage of patients referred by nephrologists with detailed baseline information on diagnosis, data and laboratory treatments. In addition, we have a long follow-up period (20 years). However, one weakness is missing data for some variables (e.g. cause of mortality, medications used, body weight and patient’s food consumption). Because this is a 20-year retrospective cohort, many medical records were no longer available for access. In this sense, as these data were not found in the medical records, we did not use these data in our analysis. Furthermore, the study was performed in a point-cohort design in Brazil; so, the results on the influence of baseline indicators on patient’s survival time, in particular, may not reflect those from other countries.

## Conclusion

In summary, with the presented prediction model of the dynamics of biochemical parameter and clinical parameters at baseline (first month of admission on HD), it is possible to give a reasonably accurate estimation on the survival of patients with HD. The management of those biochemical parameter in the HD population remains challenging, given the proportion of high risk of death in patients with low levels of serum albumin, ferritin, calcium and phosphorus, evidencing the multifactorial and interrelated nature between several mechanisms that contribute to the progression of CKD, such as inflammation and malnutrition.

This prediction models of survival time can aid nephrologists in providing patients with well-founded information on their future prospects. Even this prognosis be only an absolute risk estimate, it can be reassuring for patients to know where they stand. Furthermore, this model can also guide clinical decision-making, identifying patients at high risk and allow optimization of preventive measures. Intensive intervention to reduce death of patients in the first year of RRT treatment is highly recommended, as well as outpatient nephrological care in pre-dialysis. If no known treatment is available, a nephrologist and her team could still change his approach by intensifying follow-up of the high-risk patient.

## Data Availability

The datasets used and/or analysed during the current study are available from the corresponding author on reasonable request.

## Abbreviations

ESRD: End-stage renal disease
CKD: Chronic kidney disease
RRT: Replacement renal therapy
HD: Hemodialysis
CAPD: Continuous ambulatory peritoneal dialysis
GFR: Glomerular Filtration Rate
PTH: Parathormonium
PRU: Rheumatologic profile
nPCR: Normalized catabolic protein rate
KDOQI: Renal Disease Outcome Quality Initiative
SD: Standard deviation
IQR: Interquartile range
CI: Confidence interval
OR: Odds ratio
RBC: Red blood cell count
